# Suppression of Locomotor Activity in Female C57Bl/6J Mice Treated with Interleukin-1β: Investigating a Method for the Study of Fatigue in Laboratory Animals

**DOI:** 10.1371/journal.pone.0140678

**Published:** 2015-10-15

**Authors:** David R. Bonsall, Hyunji Kim, Catherine Tocci, Awa Ndiaye, Abbey Petronzio, Grace McKay-Corkum, Penny C. Molyneux, Thomas E. Scammell, Mary E. Harrington

**Affiliations:** 1 Neuroscience Program, Smith College, Northampton, MA 01063, United States of America; 2 Department of Neurology, Beth Israel Deaconess Medical Center, Boston, MA 02215, United States of America; University of Texas Southwestern Medical Center, UNITED STATES

## Abstract

Fatigue is a disabling symptom in patients with multiple sclerosis and Parkinson’s Disease, and is also common in patients with traumatic brain injury, cancer, and inflammatory disorders. Little is known about the neurobiology of fatigue, in part due to the lack of an approach to induce fatigue in laboratory animals. Fatigue is a common response to systemic challenge by pathogens, a response in part mediated through action of the pro-inflammatory cytokine interleukin-1 beta (IL-1β). We investigated the behavioral responses of mice to IL-1β. Female C57Bl/6J mice of 3 ages were administered IL-1β at various doses i.p. Interleukin-1β reduced locomotor activity, and sensitivity increased with age. Further experiments were conducted with middle-aged females. Centrally administered IL-1β dose-dependently reduced locomotor activity. Using doses of IL-1β that caused suppression of locomotor activity, we measured minimal signs of sickness, such as hyperthermia, pain or anhedonia (as measured with abdominal temperature probes, pre-treatment with the analgesic buprenorphine and through sucrose preference, respectively), all of which are responses commonly reported with higher doses. We found that middle-aged orexin^-/-^ mice showed equivalent effects of IL-1β on locomotor activity as seen in wild-type controls, suggesting that orexins are not necessary for IL-1β -induced reductions in wheel-running. Given that the availability and success of therapeutic treatments for fatigue is currently limited, we examined the effectiveness of two potential clinical treatments, modafinil and methylphenidate. We found that these treatments were variably successful in restoring locomotor activity after IL-1β administration. This provides one step toward development of a satisfactory animal model of the multidimensional experience of fatigue, a model that could allow us to determine possible pathways through which inflammation induces fatigue, and could lead to novel treatments for reversal of fatigue.

## Introduction

Fatigue is a symptom associated with many neurological disorders, particularly multiple sclerosis and Parkinson’s Disease. In the general population, the prevalence of fatigue increases with age, particularly in women and is estimated to cost U.S. employers $136 billion annually through reduced productivity [1]. In diseases such as multiple sclerosis, prevalence may be as high as 85% [2]. Currently, researchers have little understanding of the neurobiological basis of disease-associated fatigue, and clinicians have few effective treatments to offer patients.

Fatigue is common with inflammation [3–5]. In response to an immune challenge, the organism mounts behavioral and physiological actions, which have been collectively termed “sickness behaviors”. These include fatigue, fever, social withdrawal, anorexia, anhedonia and muscle-ache [6]. Prior research has effectively utilized pathogens and related antigens that act on Toll-like receptors (TLRs) to bring about sickness behaviors. These include the bacterial endotoxin lipopolysaccharide (LPS) [7], viral mimetics such as polyinosinic:polycytidylic acid (Poly I:C)[8] and inoculation with the parasite *Trypanosoma brucei* [9]. Sickness behaviors are mediated through multiple pro-inflammatory cytokines, including interleukin-1β (IL-1β), interleukin-6, tumor necrosis factor-α and interferon [6].

How might one study fatigue separate from other sickness behaviors? We began these studies with the goal of trying to separate immune activation effects on locomotor activity from the wider array of sickness behaviors. We focused on wheel running behavior because it is a voluntary activity that can be intense and thus effortful, and might tap both lethargy as well as altered motivation associated with the symptom of fatigue. In animal studies, immune system activation or treatment with chemotherapy causes a reduction in wheel-running as well as general locomotor activity [8–12]. Some evidence suggests that IL-1β in particular is linked to the reduction of activity. Peripherally administered IL-1β reduces locomotor activity in mice [13,14] through activity on CNS endothelial cells [15,16]. Disruption of IL-1β signaling in the CNS attenuates LPS effects on locomotor activity [17,18]. Furthermore, central administration of an interleukin-1 receptor antagonist or IL-1β neutralizing antibody attenuates the reduction in wheel-running activity caused by peripheral administration of IL-1β or Poly I:C [12,19,20].

Clinical studies support the direction of an inflammation-based animal model of fatigue. Cytokine induction in healthy individuals causes feelings of fatigue [21]. Cancer-related fatigue is correlated with markers of inflammation (C-reactive protein, IL1-receptor antagonist, IL-6) [22,23]. Both fatigue and inflammation increase with age [24]. Although an animal model will not replicate all the complex symptoms and correlates associated with fatigue in clinical populations [25], such a model may allow us to determine specific mechanisms by which inflammation induces fatigue in clinical populations.

It is still unclear in which brain regions immune activation induces suppression of locomotor activity. One hypothesis is that orexin-containing neurons of the lateral hypothalamus play an important role. Mice lacking orexins have reduced running-wheel activity [26]. Both inflammation and chemotherapy decrease orexin (hypocretin) neuron activity, with effects on wheel-use reversed by central administration of orexin-A [7,27,28].

In the studies reported below, we determined that IL-1β can reduce locomotor activity in an age- and dose-dependent manner. We focused our subsequent studies on middle-aged female mice because our initial studies showed that young mice were less sensitive to these effects of IL-1β and because fatigue is most highly reported in middle-aged women. To determine the possibility of this effect on locomotor behavior being separable from expected effects on a range of sickness behaviors, we conducted further studies with one dose to measure its effects on sleep, body temperature, anhedonia and pain in middle-aged female mice. To determine if IL-1β effects are mediated by orexin, we tested the hypothesis that orexin gene knockout mice will not show further reduced wheel-running activity after IL-1β. Given the limited number of clinical treatments available to treat fatigue [2], we subsequently tested two potential pharmacological treatments to reverse these effects on locomotor activity. These included the CNS stimulant methylphenidate and a drug commonly prescribed to alleviate daytime sleepiness, modafinil. These drugs are both thought to block the reuptake of dopamine, a neurotransmitter strongly implicated in the signaling of motivated behaviors [29,30]. Effectiveness of either of these compounds would make sense in light of a current theory of dopamine circuit dysfunction underlying fatigue [31].

## Methods

### Animals

The Smith College Animal Care & Use Committee approved these studies, which were conducted in a facility certified by the Association for Assessment and Accreditation of Laboratory Animal Care (AAALAC). Female C57Bl/6J mice were bred in-house at Smith College under a 12:12 hour light-dark cycle and provided with food (2014 Teklad Global 14% Protein Diet; Harlan Laboratories) and water *ad libitum*, with Harlan Tek-Fresh bedding. Our first experiment used young (3–5 months), middle-aged (6–12 months) and aged (18–24 months) mice; all subsequent experiments were conducted using middle-aged (6–12 months) mice. Heterozygous prepro-orexin knockout mice backcrossed more than 6 generations to C57Bl/6J [32] were crossed to produce homozygous prepro-orexin knockout and wildtype littermates. Offspring were genotyped as previously described [26,32] by PCR, using primers for the wildtype allele, 5’-GACGACGGCCTCAGACTTCTTGGG, 3’-TCACCCCCTTGGGATAGCCCTTCC (1.5kb product) and the mutant knockout allele, 5’-TAGTTGCCAGCCATCTGTTG, 3’-ACTCTCCACCCACAGACAGG (2.1kb product). These mice were group housed until age 6 months.

### Home cage behavioral measures

When animals reached the appropriate age range, they were housed individually in a cage (483mm x 267mm x 152mm, AnCare Corp. Bellmore, NY, USA) provided with a running wheel (120mm diameter) and motion sensor (K-940, Visonic, Bloomfield, CT, USA) for a minimum of two weeks to record daily wheel revolutions and general locomotor behavior respectively. Wheel and motion activity were collected in 1-minute bins using ClockLab Data Collection Software (Actimetrics Inc., Wilmette, IL, USA). Once activity levels were stable, mice were recorded for an additional 5 days to provide baseline activity in wheel revolutions and general locomotion prior to administration of IL-1β. A 5-day baseline was chosen to adjust for variability associated with the estrous cycle in females. On day 6, mice were injected i.p. 30 minutes before lights off (ZT11.5) with recombinant mouse IL-1β (carrier-free, BioLegend, San Diego, CA, USA) at doses ranging from 0–24μg/kg in 0.1ml volume. Control mice received a vehicle injection of 0.1ml pyrogen-free 0.9% saline (Besse Medical, Cincinnati, OH, USA). Activity recorded post-IL-1β was analyzed as a percentage of the mean baseline activity for each animal and as absolute counts for time course analysis. Subjects received a maximum of two treatments, limited to one vehicle and one IL-1β dose, separated by a minimum 8-day washout period. Mice were weighed before and after procedures (approx. every 10 days).

### I.C.V. administration of IL-1β

To administer IL-1β directly to the brain, middle-aged female mice were surgically implanted with a stainless steel guide cannula (22 gauge, Plastics One, Roanoke, VA, USA) stereotaxically directed at the lateral ventricle. Mice were administered 0.05mg/kg buprenorphine (Reckitt Benckiser Pharmaceuticals Inc., Richmond, VA, USA) and 5mg/kg ketoprofen (Fort Dodge Laboratories, Fort Dodge, IA, USA) analgesics subcutaneously 30 minutes prior to surgery. Under isoflurane anesthetic, the guide cannula was positioned relative to bregma (-0.4mm anterior-posterior, +1mm lateral and -3mm ventrally) and secured with a single screw anchor and dental cement (GC Fuji Plus, Patterson Dental, Rocky Hill, CT, USA). Mice were left to recover individually housed, with a dummy cap (Plastics One, Roanoke, VA, USA) preventing obstruction of the guide cannula for 7 days. Mice were closely monitored during recovery period with analgesics administered as needed. After recovery, a running wheel was placed into the animal’s home cage. Mice were given two weeks to habituate to the running-wheel and produce stable wheel-running activity. After a 5-day baseline, mice were injected i.c.v. 30 minutes before lights off with 2, 10, or 50ng recombinant mouse IL-1β (carrier-free, BioLegend, San Diego, CA, USA) prepared in artificial cerebrospinal fluid (aCSF; 2.5mM CaCl2, 124mM NaCl, 3.3mM KCl, 1.2mM KH2PO4, 1mM MgSO4, 25.5mM NaHCO3, 10mM D-Glucose, 1.14mM Ascorbic Acid) in a volume of 500nL (n = 5–7 per dose). Doses were chosen based on previous studies [33,34]. Control mice received 500nL aCSF i.c.v.

### Novel environment behavioral measures

Activity levels of middle-aged female mice were measured in a novel environment after treatment with 14μg/kg IL-1β (n = 5) or vehicle (n = 5). Mice were injected at ZT11.5 and placed back in their home cage. After 4hrs (ZT15.5), a time when wheel-use and general motion are both low in the home cage, mice were placed in a novel cage of equal size without nesting material under dim red light for 5 minutes to habituate and for a further 15 minutes to assess activity. Activity was recorded using ANY-maze video tracking software (San Diego Instruments, San Diego, CA, USA). Maximum speed was measured as the shortest time required to move one body length over the duration of assessment.

### Sleep analysis

To determine if IL-1β alters sleep-wake behavior, middle-aged female mice (n = 12) were individually placed in a non-invasive Piezo Sleep Sensor Quad Cage (Signal Solutions LLC, Lexington, KY, USA) as described previously [35]. These cages are equipped with a Polyvinylidine Difluoride (PVDF) sensor that detects pressure changes on the cage floor in response to motion. These pressure changes were measured using SleepStatLab acquisition software (Signal Solutions LLC, Lexington, KY, USA), which used a computer algorithm to predict episodic sleep activity every 2–4 seconds based on periodic changes in pressure recorded from the PVDF floor generated by regular breathing observed when mice were sleeping. These predictions of sleep-wake activity correlated strongly with human observations of sleep-wake behavior. Mice could be observed at times to be awake but not moving, and the algorithm correctly distinguished this state of quiet resting from sleep during our observations in pilot studies. Mice were habituated in the sleep cages for 3 days before commencement of the study. Sleep-wake predictions were collected continuously over 5 days to establish a stable baseline. On day 6, mice were injected i.p. 30 minutes before lights off (ZT11.5) with 14μg/kg IL-1β (shown to reduce activity to less than 25% baseline) or 0.9% saline vehicle. Two days after the first injection, all animals were counterbalanced to ensure they had received both vehicle and IL-1β doses.

### Abdominal temperature recordings

Peripherally administered IL-1β has been previously shown to induce hyperthermic responses in mice under non-thermoneutral conditions [36]. To determine if the doses of IL-1β that decreased locomotor activity were also inducing hyperthermia, middle-aged females were surgically implanted with abdominal G2 E-Mitters (Starr Life Sciences Corporation, Oakmont, PA, USA) under isoflurane anesthesia. Mice were administered 0.05mg/kg buprenorphine (Reckitt Benckiser Pharmaceuticals Inc., Richmond, VA, USA) and 5mg/kg ketoprofen (Fort Dodge Laboratories, Fort Dodge, IA, USA) analgesics subcutaneously 30 minutes prior to surgery and left to recover for 7 days. After 5 days of baseline, mice were given 14μg/kg IL-1β (n = 11), 16μg/kg IL-1β (n = 4), or vehicle (n = 15) at ZT11.5. Over the course of the experiment, each animal received a total of two injections, one dose IL-1β and one vehicle, with a minimum of 8 days between treatments. The order of injections was determined randomly. Probe data were detected by ER-4000 receivers placed under each cage and collected in 5-minute bins using the VitalView Data Acquisition System (Starr Life Sciences Corporation, Oakmont, PA, USA). All data were collected at an ambient temperature of 21°C.

### Effects of analgesic pre-treatment on IL-1β-induced fatigue

IL-1β may trigger pro-inflammatory responses resulting in pain during wheel-running activity [37]. To exclude the possible contribution of pain on wheel-running behavior, middle-aged female mice were pre-treated with the analgesic buprenorphine. Mice were initially recorded for 5 days of baseline activity. On day 6, mice received either buprenorphine/vehicle (n = 10), or buprenorphine/ IL-1β (n = 5). The analgesic buprenorphine was given at a dose of 0.05mg/kg subcutaneously 60 minutes before lights off (ZT11). Mice were injected with 14μg/kg IL-1β i.p. 30 minutes after buprenorphine pre-treatment (ZT11.5). Control groups received 0.9% saline as vehicle in lieu of drug.

### Sucrose preference

Middle-aged female mice (n = 19) were pre-exposed daily to a 4% sucrose solution (ThermoFisher Scientific Inc, Waltham, MA, USA) during the dark phase (ZT12-24) until an >80% preference over water was established. Once pre-exposure was complete, mice were given 14μg/kg IL-1β or vehicle i.p. at ZT11.5 and provided with a 2.5% sucrose solution and water. To eliminate side preference, sucrose placement was randomly divided between preferred and less preferred sides for each animal and switched after 6 hours.

### Pharmacological treatments

We tested the effects of two drugs that are occasionally used for the treatment of fatigue [2]. After 5 days of baseline recording, middle-aged females were co-administered with 14μg/kg IL-1β and 150mg/kg modafinil i.p. (Sigma-Aldrich, St. Louis, MO, USA, n = 6) or vehicle (0.25% methylcellulose in saline, n = 7) at ZT11.5. This dose has previously been shown to increase locomotor activity in rodents [38]. In line with the study design by Antle et al., [39], a separate cohort received methylphenidate hydrochloride (0.8–1.6mg/ml, Sigma-Aldrich, St. Louis, MO, USA) in the drinking water during the 5 days of baseline activity and for 24hrs post-IL-1β given on day 6 at ZT11.5. This concentration range has been previously shown to increase wheel-running behavior in mice [39]. Consumption was measured daily and the dose of methylphenidate (mg/kg) received during IL-1β-induced fatigue was calculated post hoc. Control animals received either methylphenidate in the drinking water and a vehicle i.p. injection on day 6 (n = 8), or drinking water and 14μg/kg IL-1β i.p. on day 6 (n = 10).

### Data analysis

Behavioral activity (wheel rotations and motion around the cage) over the 5 baseline days and treatment day was binned into 30 minute counts and exported as time-series using ClockLab Analysis Software (Actimetrics Inc., Wilmette, IL, USA). Nocturnal activity following IL-1β was calculated as a percentage of the mean 5-day baseline nocturnal activity levels. Individual bouts of wheel-running activity were defined as periods with no more than 1 minute of inactivity.

Core body temperature was corrected for activity-dependent heat generation based on methods previously reported [40]. Briefly, core body temperature over the 5-day baseline period is normalized and plotted against activity (as measured by the abdominal G2 E-mitter probe). The slope of a least-squares regression plotted through these data calculated the additional heat generated by the previous 5 minutes of activity. The activity-dependent contribution was subtracted providing an activity-independent temperature change.

Changes in baseline activity with age and pre-treatment with buprenorphine were analyzed by one-way ANOVA with Tukey-Kramer multiple comparisons post hoc tests. The effects of IL-1β over time, on core body temperature, sleep and sucrose preference were analyzed by two-way ANOVA (time x treatment) with Tukey-Kramer multiple comparisons post hoc test. Analyses of wheel-running bouts, velocity changes and effects of modafinil were performed by two-tailed Student’s t-test. The correlation between increasing methylphenidate concentrations and wheel-running activity was determined by Pearson’s correlation coefficient. For all experiments the experimental unit was an individual animal.

## Results

### IL-1β decreases wheel-running and general locomotor behaviors in a dose-and age-dependent manner

**Fig 1:**
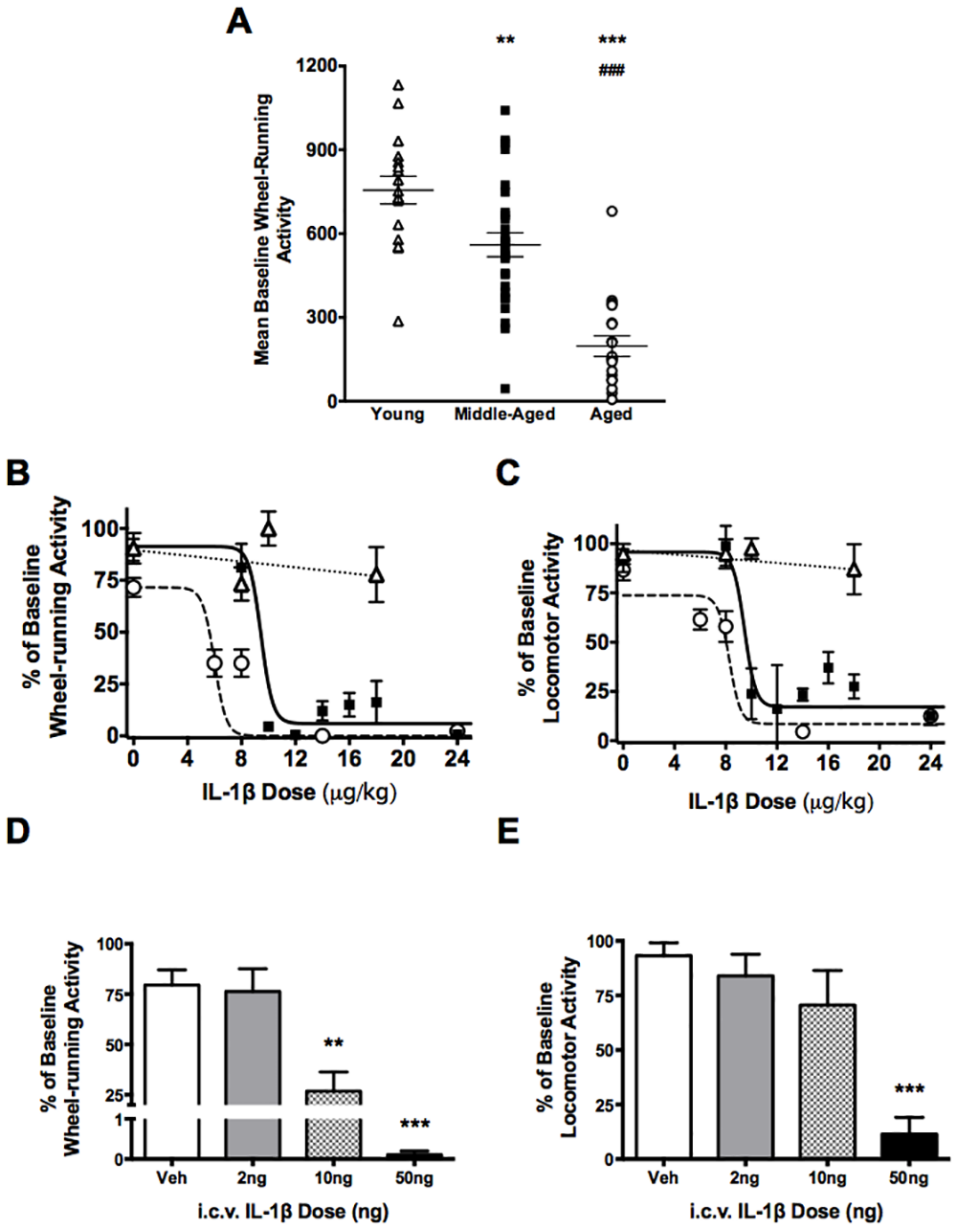
Dose- and age-dependent effects of IL-1MID: locomotor activity. (A) Spontaneous running-wheel use declines with age in female C57Bl/6 mice. Peripheral IL-1β reduced (B) wheel-running and (C) general locomotor activity in middle-aged (black squares, n = 2–15) and aged (white circles, n = 4–16) female C57Bl6 mice. IL-1β given i.p. had little effect on wheel running in young (white triangles, n = 4–8) female mice. IL-1β given centrally dose-dependently reduced (D) running-wheel and (E) general locomotor activity of middle-aged female mice (n = 5–7 per dose). Data shown as the percentage mean ± SEM 24hr post-treatment activity relative to the mean activity of the previous 5 days (‘baseline activity’), **p<0.01, *** p<0.001 differences compared to young / vehicle, ### p<0.001 differences compared to middle-aged.

Voluntary use of running wheels declined with age in female C57Bl/6J mice (Fig 1a). Analysis of baseline wheel-running activity by one-way ANOVA showed significant variation between the daily wheel use of young (3–5 months, 756±49 counts), middle-aged (6–12 months, 560±43 counts) and aged (18–24 months, 197±37 counts) female mice (F_(5,116)_ = 27.09, p<0.0001).

Increasing doses of IL-1β did not significantly attenuate wheel-running activity in young female mice within the range tested (F_(3,30)_ = 1.217, p = 0.32, one-way ANOVA). However, middle-aged and aged females showed sensitivity to IL-1β starting at 10μg/kg (95±1% decrease from baseline, F_(7,75)_ = 24.88, p<0.0001, one-way ANOVA) and 6μg/kg (65±7% decrease from baseline, F_(4,52)_ = 19.77, p<0.0001, one-way ANOVA) respectively (Fig 1b). Similar responses to IL-1β were seen in general locomotor activity of young (F_(3,30)_ = 0.219, p = 0.88, one-way ANOVA), middle-aged (76±13% decrease from baseline, F_(7,75)_ = 16.68, p<0.0001, one-way ANOVA) and aged (39±5% decrease from baseline, F_(4,52)_ = 20.84, p<0.0001, one-way ANOVA) females over the same dose range (Fig 1c).

Central administration of IL-1β directly into the lateral ventricle resulted in dose-dependent decreases in wheel-running activity in middle-aged females (Fig 1d). Doses of 10ng and 50ng IL-1β reduced wheel-running activity by 73±10% (F_(3,18)_ = 18.54, p = 0.001, one-way ANOVA) and 99±0.1% (p<0.0001) respectively. General locomotor activity was also reduced in a dose-dependent manner with 50ng IL-1β significantly suppressing activity by 88±8% (F_(3,18)_ = 8.506, p = 0.0007, one-way ANOVA, Fig 1e).

**Fig 2:**
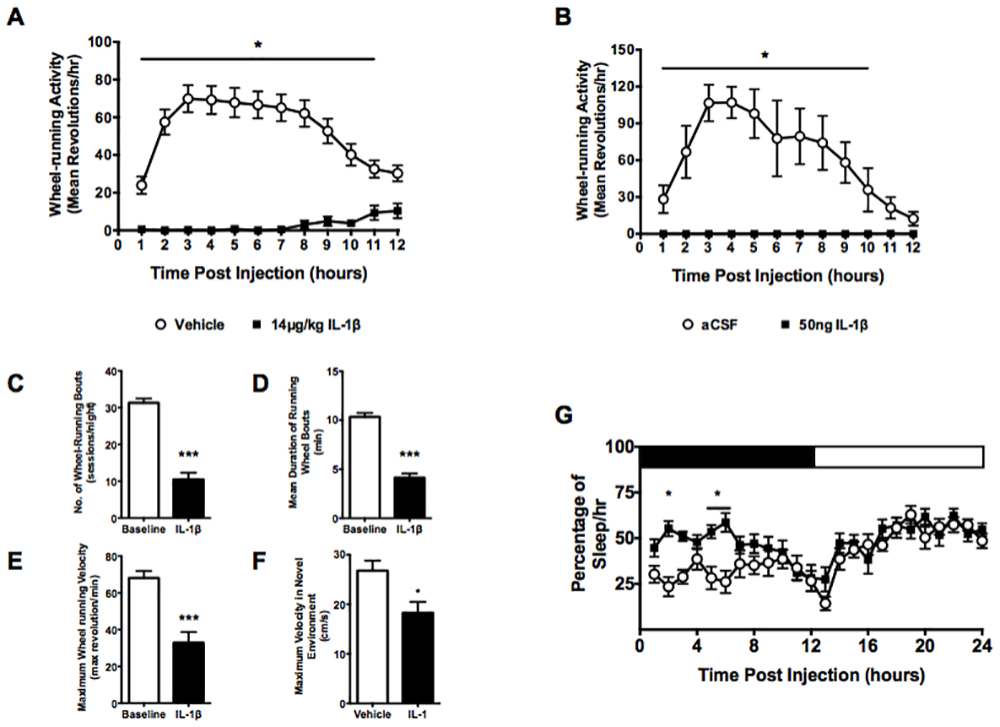
Time course of activity levels in middle-aged female mice. Administration of 14μg/kg IL-1β (black squares, n = 15) suppressed (A) wheel-running activity during the active (dark) phase compared to vehicle (white circles, n = 35). (B) Similar reductions in wheel-running activity were seen in mice given 50ng IL-1β i.c.v. (n = 5). (C) The total number of wheel-running sessions during the dark period was reduced by IL-1β, with increased fatigability (measured as decreased bout duration) (D) seen during each session as compared to baseline levels. Individual wheel-running bouts were defined as any period of wheel use with no more than 1 minute of inactivity. Mice showed reduced maximum velocity in their movement, both (E) on the wheel and (F) when placed in a novel environment. (G) IL-1β transiently increased sleep during the first 6hrs in Piezo Sleep Quad cages (n = 12). Bars show mean ± SEM. *p<0.05, ***p<0.001.

Middle-aged female mice given 14μg/kg IL-1β 30 minutes prior to lights off show suppressed wheel-running activity throughout their active phase (Fig 2a). Analysis by two-way ANOVA indicated significant effects of time (F_(11,902)_ = 16.71, p<0.0001) and treatment (F_(2,82)_ = 37.07, p<0.0001) with a significant interaction (F_(22,902)_ = 5.86, p<0.0001) on wheel-running activity. Post hoc analysis confirmed IL-1β-induced changes in wheel-running activity until 12 hours post injection, when baseline activity decreased at the end of the active phase. Central administration of 50ng IL-1β also suppressed wheel-running activity throughout the night (Fig 2b). There were significant effects of time (F_(11,132)_ = 13.79, p<0.0001), treatment (F_(2,12)_ = 36.60, p<0.0001) and interaction (F_(22,132)_ = 4.11, p<0.0001). Multiple comparisons post hoc analysis showed significantly lower activity until 11 hours post injection, when baseline activity decreased.

The suppression of locomotor activity in middle-aged female mice by IL-1β were further measured in terms of frequency and duration (as indications of stamina) as well as velocity. Mice treated with IL-1β showed a 66% reduction in the number of wheel-use sessions during the night (11±2 sessions compared to 31±1 sessions during baseline days, p<0.0001, t = 7.699, unpaired t-test, Fig 2c). A single session was concluded when there was greater than 1 minute without wheel use. IL-1β-treated mice also showed a 60% reduction in the duration of wheel-running sessions (4.14±0.4 minutes compared to 10±0.4 minutes during baseline days, p<0.0001, t = 6.596, unpaired t-test, Fig 2d) and a 51% reduction in the maximum speed of wheel revolutions (33±6 revolutions/min compared to 68±4 revolutions/min during baseline days, p<0.0001, t = 5.161, unpaired t-test, Fig 2e). The effects on speed were not restricted to the home cage and wheel-use. The maximum locomotor speed of mice placed into a novel environment 4 hours after receiving 14μg/kg IL-1β was 32% slower than vehicle treated mice (18±2cm/s compared to 27±2cm/s, p = 0.0218, t = 2.841 unpaired t-test, Fig 2f).

IL-1β has been previously shown to have somnogenic effects in rodents when administered centrally [41]. To determine whether sleepiness might reduce wheel and locomotor activity, middle-aged females were placed into non-invasive Piezo Sleep Sensor Quad cages [35]. There were significant effects of treatment (F_(1, 11)_ = 35.17, p<0.0001), time (F_(35,385)_ = 9.592, p<0.0001) and an interaction of both factors (F_(35,385)_ = 2.200, p = 0.0002, repeated measures two-way ANOVA). Post hoc analysis showed that IL-1β increased the number of sleep-associated recordings as detected by the computer algorithm. This increase was seen at 2 hours (p = 0.0004), 5 hours (p = 0.0159) and 6 hours (p = 0.0003) post injection compared to vehicle controls (Fig 2g). Over the first 6 hours post injection, vehicle control mice spent 29±2% per hour in computer-scored sleep whereas IL-1β treated mice spent 52±2% per hour (F_(1,71)_ = 36.73, p<0.0001). However, there were no significant effects of IL-1β on computer-scored sleep during the second 6 hours post injection with control mice spending 34±2% per hour and IL-1β-treated mice 40±2% per hour (p = 0.0952).

### Suppression of locomotor activity was induced independently of hyperthermia, pain or depressive-like symptoms

**Fig 3:**
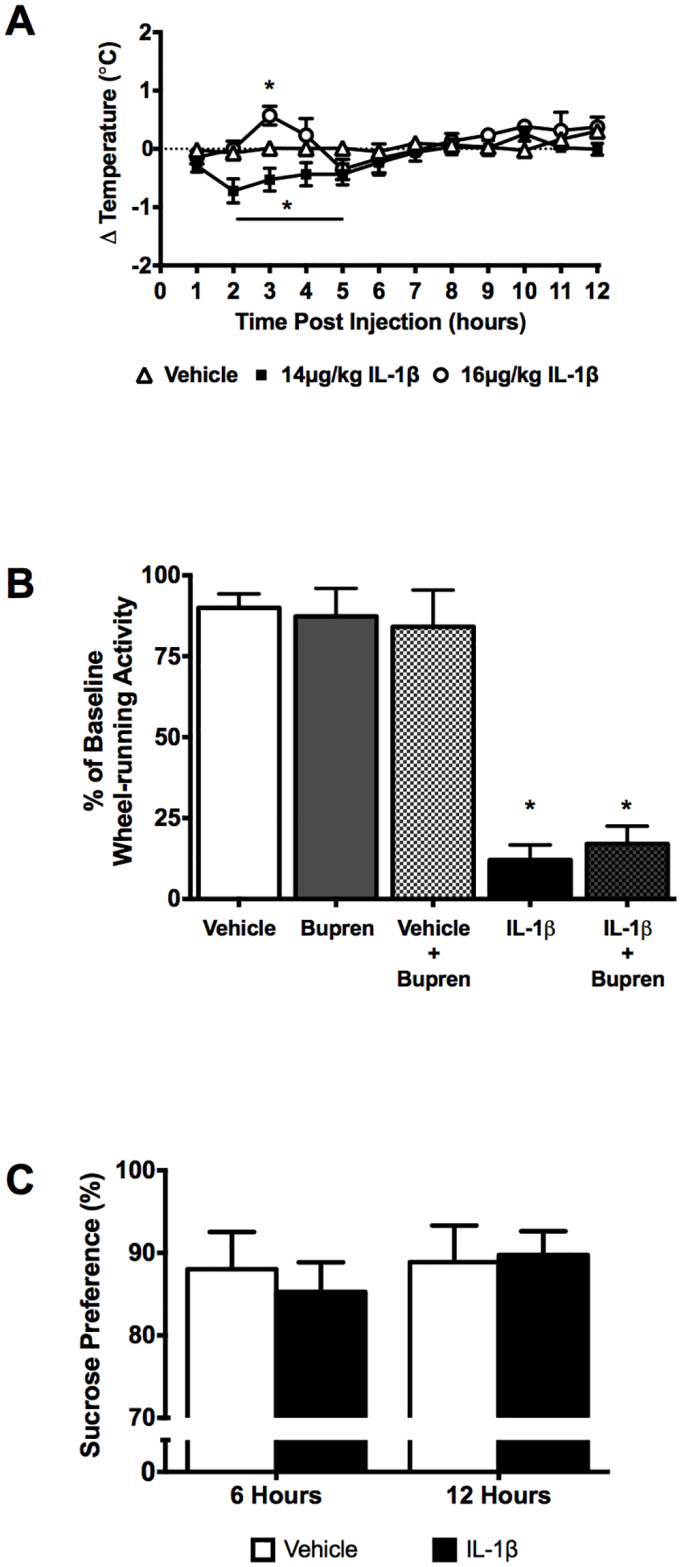
Effects of IL-1β on sickness responses. (A) Middle-aged female mice given 14μg/kg IL-1β showed mild hypothermic responses after correction for heat generated through activity (n = 11) when measured under non-thermoneutral conditions. However, higher doses of IL-1β produced transient hyperthemia (n = 4). (B) 14μg/kg IL-1β did not reduce wheel-running activity via systemic pain induction. Pre-treatment with the analgesic buprenorphine (0.05mg/kg) did not alter IL-1β –induced reductions in wheel-running activity (n = 5). Bars show mean ± SEM change in wheel-running activity over the 24hr post-treatment as a percentage of the average 5 day baseline activity. (C) Reduced wheel-running activity by IL-1β was not caused by anhedonia. Sucrose preference was measured 6hrs and 12hrs into the active (dark) phase after IL-1β treatment, with no changes in preference reported (n = 19). *p<0.05.

IL-1β is a pleiotropic cytokine linked to many sickness behaviors and responses including fever [41], pain [37] and depression [42]. Middle-aged females had lower core body temperature between 2–5 hours after treatment with 14μg/kg IL-1β (-0.4 to -0.7°C) compared to vehicle controls (Fig 3a). Temperature measures were corrected for differences in activity-dependent heat generation since IL-1β reduces locomotor activity. At 14ug/kg, a hypothermic response was seen that was not accounted for by inactivity. These measures were taken in an ambient environment of 21°C and not under thermoneutral conditions. Furthermore, it is possible that the increased sleep occurring during the early night could contribute to the hypothermic response, as sleep has been shown to reduce core body temperature [43]. Despite this cooler environment, mice administered 16μg/kg IL-1β showed a transient hyperthermic response 3 hours after injection (+0.6°C, p = 0.0415), also independent of changes in activity-generated heat. Two-way ANOVA showed significant effects of time (F_(11,297)_ = 5.932, p<0.0001), treatment (F_(2,27)_ = 4.022, p = 0.0296) as well as an interaction (F_(22,297)_ = 3.018, p<0.0001).

Subcutaneous administration of the analgesic buprenorphine prior to IL-1β treatment was given to determine if pain contributed to the suppression of locomotor activity. Buprenorphine with vehicle did not alter running-wheel activity, compared to vehicle alone (p = 0.9688, one-way ANOVA) or buprenorphine alone (p = 0.9988, one-way ANOVA) (Fig 3b). Administration of 14μg/kg IL-1β significantly reduced wheel-running activity by 88 ± 5% (p<0.0001, one-way ANOVA), which was not significantly affected by pre-treatment with buprenorphine, resulting in a 83 ± 5% attenuation by 14μg/kg IL-1β (p = 0.9955, one-way ANOVA). Buprenorphine pretreatment prior to IL-1β resulted in significantly less wheel-running activity than control or buprenorphine alone (p<0.0001).

Sucrose preference over water consumption was measured in vehicle and IL-1β treated middle-aged mice after 6 and 12 hours post injection as a measure of anhedonia (Fig 3c). Two-way repeated measures ANOVA showed no significant effects of time (F_(1,18)_ = 1.545, p = 0.2299), treatment (F_(1,18)_ = 0.06237, p = 0.8056) or a significant interaction (F_(1,18)_ = 1.001, p = 0.3303) after 6 hours (88±5% for control, 85±4% for IL-1β treated, p = 0.5033) or after 12 hours (89±4% for control, 90±3% for IL-1β, p = 0.9324).

### Orexins are not a necessary component of IL-1β-induced fatigue

**Fig 4:**
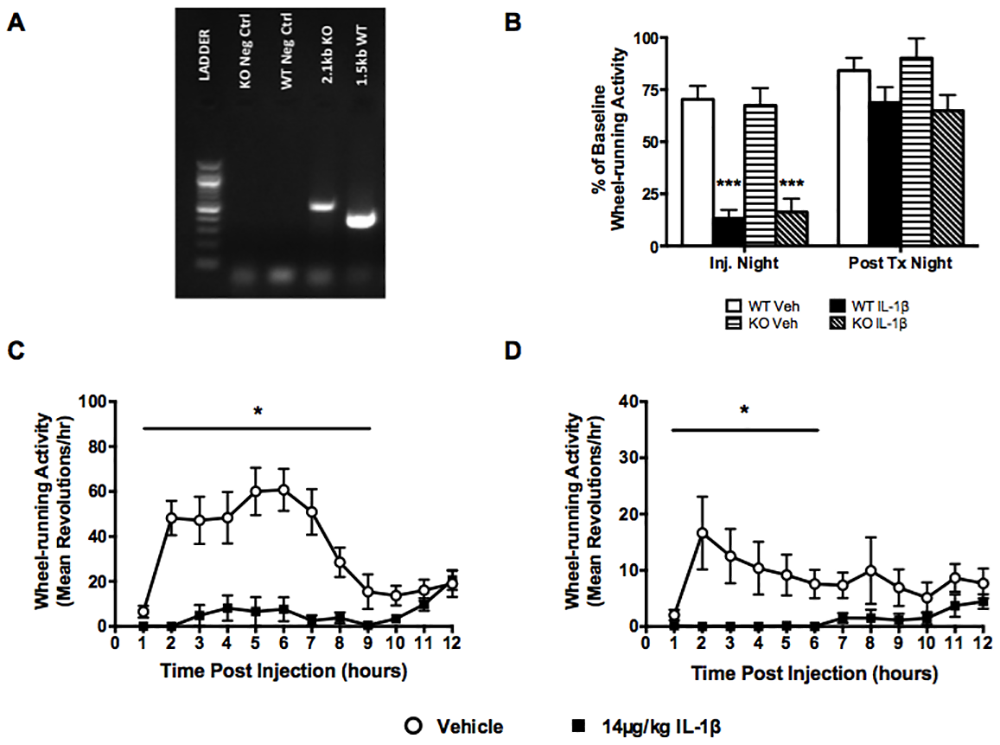
Orexins are not necessary to mediate IL-1β-induced reductions in activity. (A) Representative gel of genotyping results, distinguishing the wild-type allele (1.5kb) from the mutant prepro-orexin knockout allele (2.1kb). (B) 14μg/kg IL-1β reduced wheel-running activity in female (C) wild type and (D) prepro-orexin knockout littermates to similar levels of baseline (n = 12–14). Data expressed as mean ± SEM, *p<0.05, **p<0.01, ***p<0.001.

Decreased cellular activity of orexin-containing neurons was previously found to be associated with LPS-induced lethargy [27]. To determine if a change in orexin signaling is required to induce fatigue-like behaviors, orexin-deficient mice and their wildtype littermates were given 14μg/kg IL-1β (Fig 4). As percentages of baseline, IL-1β reduced wheel-running activity to comparable levels in both wildtype and orexin deficient mice (Fig 4b). However, when comparing total counts of baseline activity, wildtype females showed significantly more nocturnal use of the running wheel than orexin deficient mice (580±56 counts in wildtype, 146±36 counts in knockouts, p<0.0001, t = 6.268, unpaired t-test). Middle-aged female wildtype littermates showed significant effects of time (F_(11,429)_ = 22.99, p<0.0001), treatment (F_(2,39)_ = 29.94, p<0.0001) and interaction (F_(22,429)_ = 7.187, p = 0.0001) with decreased wheel-running activity for the first 9 hours post injection (Fig 4c). Middle-aged orexin-deficient females also showed significantly decreased wheel-running activity after IL-1β treatment during the first 6 hours (Fig 4d), with significant effects of time (F_(11,363)_ = 2.610, p = 0.0033) and treatment (F_(2,33)_ = 6.554, p = 0.0040) with the interaction term not making our cut-off of p<.05 but perhaps indicating a trend in that direction (F_(22,363)_ = 1.504, p = 0.0687).

### Current pharmacological treatments have limited success in alleviating fatigue

**Fig 5:**
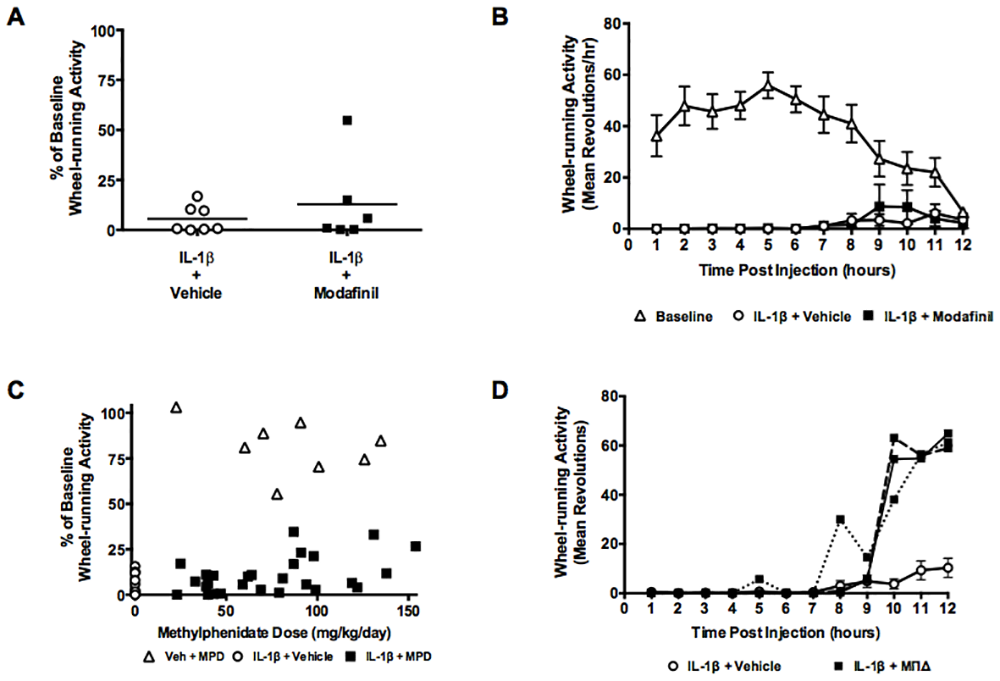
Effects of stimulants on IL-1β-induced reductions in locomotor activity. (A, B) Pre-treatment with 150mg/kg modafinil did not significantly attenuate the effects of IL-1β on wheel-running in middle-aged females (n = 6–7). (C) Increasing doses of methylphenidate, provided in the drinking water was correlated with increased wheel-running activity after IL-1β. (A-C) IL-1β + vehicle (white circles), IL-1β + treatment (black squares), methylphenidate + vehicle (white triangles). (D) Example time courses of wheel-running activity for three mice, dosed with methylphenidate, with post-IL-1β activity levels greater than 25% of baseline. IL-1β + vehicle (white circles), IL-1β + methylphenidate (black squares), different line styles indicate different animals.

Among the few pharmacological options available for patients suffering from fatigue with no identifiable cause are the stimulants modafinil and methylphenidate, which have limited success at alleviating the symptom [44,45]. Modafinil given alongside IL-1β did not improve the reduced wheel-running activity caused by IL-1β alone (13±9% of baseline compared to 6±3% by IL-1β alone, p = 0.4035, t = 0.8689, unpaired t-test) (Fig 5a). Analysis of the time course by two-way ANOVA (Fig 5b) confirmed a significant treatment effect of IL-1β (F_(2,21)_ = 85.10, p<0.0001) and interaction with time (F_(22,231)_ = 4.013, p<0.0001) but no main effect of time (F_(11,231)_ = 1.643, p = 0.0879). Post hoc analysis confirmed no improvement with modafinil co-administration.

Methylphenidate added to the drinking water provided a self-medicating regime. Daily dose was variable across subjects, dependent on both drug concentration and individual consumption of water (Fig 5c). When binned as 25mg/kg/day increments, one-way ANOVA confirmed a significant effect of treatment with 125-150mg/kg/day (22±11% compared with 6±2% for untreated fatigued mice, F_(5,25)_ = 2.928, p = 0.0325). Analysis by Pearson’s correlation results in a coefficient r = 0.4619, p = 0.0040, suggesting that with increasing concentrations of methylphenidate, wheel-running activity was partially restored in some animals. Hourly time courses for these individuals indicate earlier restoration of activity towards the end of the night, approximately 10hrs post injection (Fig 5d).

## Discussion

Our characterization of the effects of different doses of IL-1β in mice of different ages exemplifies the critical importance of these variables in studies of cytokine-induced suppression of locomotor activity in mice. Young mice were uniformly resistant to these effects in the doses tested. After this initial characterization, we chose to conduct the bulk of our further studies with middle-aged (6-12mos) female mice. With this standardization of the subject characteristics, dose becomes the defining variable that determines if the mouse shows effects solely on locomotor activity, or (as dose is increased) if the mouse also shows other aspects of sickness behaviour, such as hyperthermia. We expect that with higher doses IL-1β would induce the full range of sickness behaviors, and a key contribution of our work is the characterization of the dose-response curve for effects on locomotor activity. Our initial work followed prior studies [13,46,47] in administering a set nanogram amount of IL-1β per animal, but our analysis and later studies demonstrated that we are better able to predict responses when the dose is adjusted for the animal’s body weight. When given 14μg/kg IL-1β, middle-aged females showed reduced locomotor activity throughout the night. Using the piezo sleep quad cage system, sleep-associated changes in breathing and motion were observed in the first half of the night; our methods cannot distinguish individual sleep stages, but IL-1β has been previously reported to increase non-REM sleep [48]. We did not see other significant effects of this dose of IL-1β such as pain-induction (as determined by pre-treatment with an analgesic) or depressive-like symptoms (assessed through sucrose preference) that may otherwise have accounted for the decrease in locomotor activity (Fig 3b and 3c)[49,50]. However, it is important to note that these effects might be present at a level that our tests were not sensitive enough to measure, and that with increasing doses of IL-1β, these symptoms would be expected to emerge alongside the reductions in locomotor activity. Neuroinflammation is common in many neurological disorders that share the symptom of reductions in locomotor activity [5]. We have shown here that direct administration of IL-1β into the brain may suppress behavioral activity in a similar dose-dependent manner as that produced by peripheral IL-1β, supporting a centrally mediated mechanism of induction [46,51].

Fatigue, as described by patients, is a multifaceted symptom, with cognitive and emotional aspects as well as reductions in locomotor activity [52]. To investigate fatigue using laboratory animals, other researchers have also found measures of wheel-running useful [53–55]; it remains to be determined if these lab animals express cognitive and/or emotional aspects of fatigue reported in human populations. IL-1β treated animals manifest not only an overall reduction in wheel-use, but changes in the pattern of wheel-use, including shorter bouts of exertion (Fig 2c–2f). These changes are common measures of increased fatigability, an important aspect of the symptom of fatigue [2].

A key rationale for these studies was to develop a method to allow determination of neurobiological mechanisms underlying inflammation-induced reduction in locomotor activity. Based on prior research suggesting a critical role for the orexin-expressing lateral hypothalamic neurons [7,27,28], we predicted that orexin-deficient mice would not show reduced locomotor activity following administration of IL-1β. We replicated prior findings that the prepro-orexin knockout mice have reduced wheel-use [26]; yet, despite this lowered basal level, when given IL-1β these mice showed further reductions in wheel-use, similar in proportion to that shown by wildtype littermates. Therefore, our experiment suggests that orexins are not, by themselves, a necessary component in IL-1β cytokine signalling decreased locomotor activity. On the other hand, it remains possible that these lateral hypothalamic neurons may play a role in signalling inflammation-induced changes in locomotor activity. Inflammation and chemotherapy- induced lethargy was associated with reduced cellular activity within orexin-containing neurons of the lateral hypothalamus [7,27,28]. These neurons are still present in the orexin-deficient mice used in the current study, and have been shown to contain several other neurotransmitters including dynorphin and glutamate [56,57], that may be involved in inflammation-induced lethargy. Our experiment has not ruled out an orexin-independent role for these neurons, nor a non-essential role for orexins in mediating reduced locomotor activity. These experiments have demonstrated that IL-1β can induce suppression of locomotor activity even in mice that are more sedentary, as inferred by the baseline reduced wheel use.

In general, current medications produce little improvement in fatigue. We tested two commonly prescribed stimulants, modafinil and methylphenidate, tested for effect in patients with either Parkinson’s Disease suffering from fatigue [2], or cancer-related fatigue [2,58]. Patients being treated with modafinil typically report improvement in excessive daytime sleepiness, measured by the Epworth Sleepiness Score, but no improvement in self-reporting of subjective fatigue (reviewed by [59]). We found no change in IL-1β–induced decreased locomotor activity with co-administration of modafinil. We have not determined whether modafinil attenuated IL-1β -induced increases in sleep, but our data concur with clinical findings that modafinil is ineffective in treating fatigue of this extent [45,60]. One study of cancer-related fatigue showed modafinil improved self-reported fatigue scores, but only in patients classified as having severe fatigue prior to the start of the study, with no improvement in mild or moderate patients [61]. The second treatment we tested was methylphenidate. Methylphenidate has been shown to improve self-report scores of fatigue in patients with Parkinson’s Disease [62] and cancer-related fatigue [58,63], with dose-dependent effects [64]. In the current study, animals were exposed to methylphenidate in the drinking water, allowing them to self-medicate throughout the night after IL-1β-administration, limiting the need for repeated handling. Higher doses of methylphenidate significantly increased wheel-running activity after IL-1β. Animals responding to methylphenidate showed earlier recovery of wheel-running activity, but improvement with methylphenidate remained at less than 40% of baseline activity. A meta-analysis of methylphenidate use with cancer-related fatigue indicates improved benefits with longer exposure [44], offering an alternative approach for future investigation.

Methylphenidate is a stimulant that acts through blocking the reuptake of dopamine and norepinephrine, particularly relevant here in the prefrontal cortex and striatum [65,66]. There is some evidence suggesting a role for dopamine and deficits in reward signals playing a role in self-reported fatigue [31,67–69], and in effort-based tasks in laboratory animals [30]. Dopamine D_2_ receptor antagonists reduce effort-based reward-seeking behaviors, which can be alleviated in part by an adenosine A_2A_ antagonist [70]. Recent work by Nunes et al. [71] has shown that IL-1β can decrease effort-based motivational responses, which are restored by treatment with the same adenosine A_2A_ antagonist, hypothesized to be acting through potentiating dopamine signalling. Research using animal models of chronic pain indicates that changes in motivation to perform effortful tasks, a component of fatigue, is mediated in part by the neuropeptide galanin driving neural plasticity in the dopamine D2 receptor-expressing nucleus accumbens neurons [72]. In addition to dopamine, changes in histamine and serotonergic signalling may also contribute to the mechanism of immune-mediated decreased locomotor activity. Gaykema et al. [73] described a reduction in several motivated behaviors in rats following LPS. These reductions were associated with attenuated c-fos induction within histaminergic neurons of the ventral tuberomammillary nucleus. Katafuchi et al. [74] reported increased expression of the serotonin transporter, together with reduced extracellular serotonin within the prefrontal cortex following treatment with Poly I:C, suggesting a role for the 5HT_1A_ in mediating aspects of immune-activation reduced locomotor activity.

### General comments

Fatigue is predominantly a subjective sensation, but it is accompanied by behavioural changes that should be able to be studied in mouse models. Fatigue is most prevalent in middle-aged women, and much of our research focuses on middle-aged female mice. Still, fatigue occurs in people of various ages and genders [75], and mouse models should thus examine mice of various ages and both sexes as the underlying mechanisms may vary. For example, immune system responsiveness changes with age; increased levels of circulating cytokines in the serum are found with age. Life history can impact these responses, as is shown from studies detailing how prior immune challenges can alter the immune system, for example, “priming” an exaggerated response to IL-1β [76]. Neuro-inflammatory mechanisms for pain sensitivity vary between sexes [77], as may similar pathways underlying fatigue. Individuals vary in their susceptibility to fatigue, and we expect genetics would play a role as well. Studies using just one inbred strain of mice, as ours did, limits variation due to genetics but runs the risk of missing samples from individuals within the larger population that are more sensitive to these effects. Further studies using mice could test outbred strains.

We focused on responses to IL-1β, given evidence linking this cytokine to fatigue. There is also good evidence linking several other cytokines to fatigue. For example, leucocytes from cancer patients with post-treatment fatigue showed increased expression from both IL-1β and IL-6 genes [78]. Patients with chronic fatigue syndrome/myalgic encephalitis show increases in both IL-1β and IL-6 [79]. In a sample of cancer patients prior to surgery, fatigue correlated with levels of IL-6 [80]; another study reported that fatigue that follows cancer treatment correlated with levels of IL-6 [23,81]. Cancer patients that show improvement in fatigue show decreases in IL-6 [82]. Thus, further studies might examine IL-6 as a potential cytokine that can alter behaviour of laboratory animals in a manner related to these patient populations. Models using different approaches to induce inflammation might allow insight into specific mechanisms able to mediate changes in behaviour and motivation as might underlie the experience of fatigue.

The need for an animal model for research related to fatigue is clear, given our lack of insight into the underlying biology and the lack of effective treatments. Here, we have ruled out a necessary role for the orexins in the IL1-induced decrease of locomotor activity. We have also demonstrated that methylphenidate treatment allowed some improvement in wheel-running activity, suggesting a possible role for dopaminergic signalling. While the precise mechanisms and brain areas involved in mediating fatigue remain unclear, the development of new tools that allow for its study will help address these questions in the future.

## References

[1] Ricci JA, Chee E, Lorandeau AL, Berger J. Fatigue in the U.S. workforce: prevalence and implications for lost productive work time. J Occup Environ Med Am Coll Occup Environ Med. 2007;49: 1–10. 10.1097/01.jom.0000249782.60321.2a 17215708

[2] Finsterer J, Mahjoub SZ. Fatigue in Healthy and Diseased Individuals. Am J Hosp Palliat Care. 2013;31: 562–575. 10.1177/1049909113494748 23892338

[3] Fletcher MA, Zeng XR, Barnes Z, Levis S, Klimas NG. Plasma cytokines in women with chronic fatigue syndrome. J Transl Med. 2009;7: 96 10.1186/1479-5876-7-96 19909538 PMC2779802

[4] Stringer EA, Baker KS, Carroll IR, Montoya JG, Chu L, Maecker HT, et al Daily cytokine fluctuations, driven by leptin, are associated with fatigue severity in chronic fatigue syndrome: evidence of inflammatory pathology. J Transl Med. 2013;11: 93 10.1186/1479-5876-11-93 23570606 PMC3637529

[5] Lindqvist D, Hall S, Surova Y, Nielsen HM, Janelidze S, Brundin L, et al Cerebrospinal fluid inflammatory markers in Parkinson’s disease—associations with depression, fatigue, and cognitive impairment. Brain Behav Immun. 2013;33: 183–189. 10.1016/j.bbi.2013.07.007 23911592

[6] McCusker RH, Kelley KW. Immune–neural connections: how the immune system’s response to infectious agents influences behavior. J Exp Biol. 2013;216: 84–98. 10.1242/jeb.073411 23225871 PMC3515033

[7] Gaykema RPA, Goehler LE. Lipopolysaccharide challenge-induced suppression of Fos in hypothalamic orexin neurons: their potential role in sickness behavior. Brain Behav Immun. 2009;23: 926–930. 10.1016/j.bbi.2009.03.005 19328847 PMC2792632

[8] Katafuchi T, Kondo T, Yasaka T, Kubo K, Take S, Yoshimura M. Prolonged effects of polyriboinosinic:polyribocytidylic acid on spontaneous running wheel activity and brain interferon-alpha mRNA in rats: a model for immunologically induced fatigue. Neuroscience. 2003;120: 837–845. 12895523 10.1016/s0306-4522(03)00365-8

[9] Chao CC, DeLaHunt M, Hu S, Close K, Peterson PK. Immunologically mediated fatigue: a murine model. Clin Immunol Immunopathol. 1992;64: 161–165. 1643746 10.1016/0090-1229(92)90194-s

[10] Smith LB, Leo MC, Anderson C, Wright TJ, Weymann KB, Wood LJ. The role of IL-1β and TNF-α signaling in the genesis of cancer treatment related symptoms (CTRS): a study using cytokine receptor-deficient mice. Brain Behav Immun. 2014;38: 66–76. 10.1016/j.bbi.2013.12.022 24412646 PMC3989411

[11] Zombeck JA, Fey EG, Lyng GD, Sonis ST. A clinically translatable mouse model for chemotherapy-related fatigue. Comp Med. 2013;63: 491–497. 24326224 PMC3866991

[12] Ifuku M, Hossain SM, Noda M, Katafuchi T. Induction of interleukin-1β by activated microglia is a prerequisite for immunologically induced fatigue. Eur J Neurosci. 2014;40: 3253–3263. 10.1111/ejn.12668 25040499

[13] Anisman H, Gibb J, Hayley S. Influence of continuous infusion of interleukin-1beta on depression-related processes in mice: corticosterone, circulating cytokines, brain monoamines, and cytokine mRNA expression. Psychopharmacology (Berl). 2008;199: 231–244. 10.1007/s00213-008-1166-z 18491079

[14] Skelly DT, Hennessy E, Dansereau M-A, Cunningham C. A systematic analysis of the peripheral and CNS effects of systemic LPS, IL-1β, [corrected] TNF-α and IL-6 challenges in C57BL/6 mice. PloS One. 2013;8: e69123 10.1371/journal.pone.0069123 23840908 PMC3698075

[15] Ridder DA, Lang M-F, Salinin S, Röderer J-P, Struss M, Maser-Gluth C, et al TAK1 in brain endothelial cells mediates fever and lethargy. J Exp Med. 2011;208: 2615–2623. 10.1084/jem.20110398 22143887 PMC3244031

[16] Matsuwaki T, Eskilsson A, Kugelberg U, Jönsson J-I, Blomqvist A. Interleukin-1β induced activation of the hypothalamus-pituitary-adrenal axis is dependent on interleukin-1 receptors on non-hematopoietic cells. Brain Behav Immun. 2014;40: 166–173. 10.1016/j.bbi.2014.03.015 24681250

[17] Layé S, Bluthé RM, Kent S, Combe C, Médina C, Parnet P, et al Subdiaphragmatic vagotomy blocks induction of IL-1 beta mRNA in mice brain in response to peripheral LPS. Am J Physiol. 1995;268: R1327–1331. 7771597 10.1152/ajpregu.1995.268.5.R1327

[18] Harden LM, du Plessis I, Roth J, Loram LC, Poole S, Laburn HP. Differences in the relative involvement of peripherally released interleukin (IL)-6, brain IL-1β and prostanoids in mediating lipopolysaccharide-induced fever and sickness behavior. Psychoneuroendocrinology. 2011;36: 608–622. 10.1016/j.psyneuen.2010.09.003 20926198

[19] Kent S, Bluthe RM, Dantzer R, Hardwick AJ, Kelley KW, Rothwell NJ, et al Different receptor mechanisms mediate the pyrogenic and behavioral effects of interleukin 1. Proc Natl Acad Sci U S A. 1992;89: 9117–9120. 1409612 10.1073/pnas.89.19.9117 PMC50076

[20] Yamato M, Tamura Y, Eguchi A, Kume S, Miyashige Y, Nakano M, et al Brain interleukin-1β and the intrinsic receptor antagonist control peripheral Toll-like receptor 3-mediated suppression of spontaneous activity in rats. PloS One. 2014;9: e90950 10.1371/journal.pone.0090950 24621600 PMC3951245

[21] Späth-Schwalbe E, Hansen K, Schmidt F, Schrezenmeier H, Marshall L, Burger K, et al Acute effects of recombinant human interleukin-6 on endocrine and central nervous sleep functions in healthy men. J Clin Endocrinol Metab. 1998;83: 1573–1579. 10.1210/jcem.83.5.4795 9589658

[22] Bower JE, Ganz PA, Tao ML, Hu W, Belin TR, Sepah S, et al Inflammatory biomarkers and fatigue during radiation therapy for breast and prostate cancer. Clin Cancer Res Off J Am Assoc Cancer Res. 2009;15: 5534–5540. 10.1158/1078-0432.CCR-08-2584 PMC2884979 19706826

[23] Liu L, Mills PJ, Rissling M, Fiorentino L, Natarajan L, Dimsdale JE, et al Fatigue and sleep quality are associated with changes in inflammatory markers in breast cancer patients undergoing chemotherapy. Brain Behav Immun. 2012;26: 706–713. 10.1016/j.bbi.2012.02.001 22406004 PMC3372667

[24] Franceschi C, Bonafè M. Centenarians as a model for healthy aging. Biochem Soc Trans. 2003;31: 457–461. 10.1042/bst0310457 12653662

[25] Bower JE, Ganz PA. Symptoms: Fatigue and Cognitive Dysfunction. Adv Exp Med Biol. 2015;862: 53–75. 10.1007/978-3-319-16366-6_5 26059929

[26] España RA, McCormack SL, Mochizuki T, Scammell TE. Running promotes wakefulness and increases cataplexy in orexin knockout mice. Sleep. 2007;30: 1417–1425. 18041476 10.1093/sleep/30.11.1417 PMC2082091

[27] Grossberg AJ, Zhu X, Leinninger GM, Levasseur PR, Braun TP, Myers MG, et al Inflammation-induced lethargy is mediated by suppression of orexin neuron activity. J Neurosci Off J Soc Neurosci. 2011;31: 11376–11386. 10.1523/JNEUROSCI.2311-11.2011 PMC3155688 21813697

[28] Weymann KB, Wood LJ, Zhu X, Marks DL. A role for orexin in cytotoxic chemotherapy-induced fatigue. Brain Behav Immun. 2014;37: 84–94. 10.1016/j.bbi.2013.11.003 24216337 PMC3951615

[29] Zolkowska D, Jain R, Rothman RB, Partilla JS, Roth BL, Setola V, et al Evidence for the Involvement of Dopamine Transporters in Behavioral Stimulant Effects of Modafinil. J Pharmacol Exp Ther. 2009;329: 738–746. 10.1124/jpet.108.146142 19197004 PMC2672878

[30] Salamone JD, Cousins MS, Bucher S. Anhedonia or anergia? Effects of haloperidol and nucleus accumbens dopamine depletion on instrumental response selection in a T-maze cost/benefit procedure. Behav Brain Res. 1994;65: 221–229. 7718155 10.1016/0166-4328(94)90108-2

[31] Dobryakova E, Genova HM, DeLuca J, Wylie GR. The dopamine imbalance hypothesis of fatigue in multiple sclerosis and other neurological disorders. Front Neurol. 2015;6: 52 10.3389/fneur.2015.00052 25814977 PMC4357260

[32] Chemelli RM, Willie JT, Sinton CM, Elmquist JK, Scammell T, Lee C, et al Narcolepsy in orexin knockout mice: molecular genetics of sleep regulation. Cell. 1999;98: 437–451. 10481909 10.1016/s0092-8674(00)81973-x

[33] Spadaro F, Dunn AJ. Intracerebroventricular administration of interleukin-1 to mice alters investigation of stimuli in a novel environment. Brain Behav Immun. 1990;4: 308–322. 2092866 10.1016/0889-1591(90)90034-n

[34] Masotto C, Caspani G, De Simoni MG, Mengozzi M, Scatturin M, Sironi M, et al Evidence for a different sensitivity to various central effects of interleukin-1 beta in mice. Brain Res Bull. 1992;28: 161–165. 1596738 10.1016/0361-9230(92)90175-w

[35] Donohue KD, Medonza DC, Crane ER, O’Hara BF. Assessment of a non-invasive high-throughput classifier for behaviours associated with sleep and wake in mice. Biomed Eng Online. 2008;7: 14 10.1186/1475-925X-7-14 18405376 PMC2365952

[36] Ching S, Zhang H, Belevych N, He L, Lai W, Pu X, et al Endothelial-specific knockdown of interleukin-1 (IL-1) type 1 receptor differentially alters CNS responses to IL-1 depending on its route of administration. J Neurosci Off J Soc Neurosci. 2007;27: 10476–10486. 10.1523/JNEUROSCI.3357-07.2007 PMC6673171 17898219

[37] Ren K, Torres R. Role of interleukin-1β during pain and inflammation. Brain Res Rev. 2009;60: 57–64. 10.1016/j.brainresrev.2008.12.020 19166877 PMC3076185

[38] Ishizuka T, Murakami M, Yamatodani A. Involvement of central histaminergic systems in modafinil-induced but not methylphenidate-induced increases in locomotor activity in rats. Eur J Pharmacol. 2008;578: 209–215. 10.1016/j.ejphar.2007.09.009 17920581

[39] Antle MC, van Diepen HC, Deboer T, Pedram P, Pereira RR, Meijer JH. Methylphenidate modifies the motion of the circadian clock. Neuropsychopharmacol Off Publ Am Coll Neuropsychopharmacol. 2012;37: 2446–2455. 10.1038/npp.2012.103 PMC3445990 22763623

[40] Cambras T, Weller JR, Anglès-Pujoràs M, Lee ML, Christopher A, Díez-Noguera A, et al Circadian desynchronization of core body temperature and sleep stages in the rat. Proc Natl Acad Sci U S A. 2007;104: 7634–7639. 10.1073/pnas.0702424104 17452631 PMC1863469

[41] Opp MR, Toth LA. Somnogenic and pyrogenic effects of interleukin-1beta and lipopolysaccharide in intact and vagotomized rats. Life Sci. 1998;62: 923–936. 9496715 10.1016/s0024-3205(98)00010-1

[42] Konsman JP, Luheshi GN, Bluthé RM, Dantzer R. The vagus nerve mediates behavioural depression, but not fever, in response to peripheral immune signals; a functional anatomical analysis. Eur J Neurosci. 2000;12: 4434–4446. 11122354 10.1046/j.0953-816x.2000.01319.x

[43] Barrett J, Lack L, Morris M. The sleep-evoked decrease of body temperature. Sleep. 1993;16: 93–99. 8446841

[44] Gong S, Sheng P, Jin H, He H, Qi E, Chen W, et al Effect of methylphenidate in patients with cancer-related fatigue: a systematic review and meta-analysis. PloS One. 2014;9: e84391 10.1371/journal.pone.0084391 24416225 PMC3885551

[45] Spathis A, Fife K, Blackhall F, Dutton S, Bahadori R, Wharton R, et al Modafinil for the treatment of fatigue in lung cancer: results of a placebo-controlled, double-blind, randomized trial. J Clin Oncol Off J Am Soc Clin Oncol. 2014;32: 1882–1888. 10.1200/JCO.2013.54.4346 24778393

[46] Bluthé RM, Dantzer R, Kelley KW. Central mediation of the effects of interleukin-1 on social exploration and body weight in mice. Psychoneuroendocrinology. 1997;22: 1–11. 9141147 10.1016/s0306-4530(96)00042-x

[47] Dunn AJ, Antoon M, Chapman Y. Reduction of exploratory behavior by intraperitoneal injection of interleukin-1 involves brain corticotropin-releasing factor. Brain Res Bull. 1991;26: 539–542. 1868353 10.1016/0361-9230(91)90092-x

[48] Baker FC, Shah S, Stewart D, Angara C, Gong H, Szymusiak R, et al Interleukin 1beta enhances non-rapid eye movement sleep and increases c-Fos protein expression in the median preoptic nucleus of the hypothalamus. Am J Physiol Regul Integr Comp Physiol. 2005;288: R998–R1005. 10.1152/ajpregu.00615.2004 15604300

[49] Safieh-Garabedian B, Poole S, Allchorne A, Winter J, Woolf CJ. Contribution of interleukin-1 beta to the inflammation-induced increase in nerve growth factor levels and inflammatory hyperalgesia. Br J Pharmacol. 1995;115: 1265–1275. 7582555 10.1111/j.1476-5381.1995.tb15035.x PMC1908795

[50] Konsman JP, Veeneman J, Combe C, Poole S, Luheshi GN, Dantzer R. Central nervous action of interleukin-1 mediates activation of limbic structures and behavioural depression in response to peripheral administration of bacterial lipopolysaccharide. Eur J Neurosci. 2008;28: 2499–2510. 10.1111/j.1460-9568.2008.06549.x 19087175

[51] Anforth HR, Bluthe RM, Bristow A, Hopkins S, Lenczowski MJ, Luheshi G, et al Biological activity and brain actions of recombinant rat interleukin-1alpha and interleukin-1beta. Eur Cytokine Netw. 1998;9: 279–288. 9831177

[52] Harrington ME. Neurobiological studies of fatigue. Prog Neurobiol. 2012;99: 93–105. 10.1016/j.pneurobio.2012.07.004 22841649 PMC3479364

[53] Ottenweller JE, Natelson BH, Gause WC, Carroll KK, Beldowicz D, Zhou XD, et al Mouse running activity is lowered by Brucella abortus treatment: a potential model to study chronic fatigue. Physiol Behav. 1998;63: 795–801. 9618001 10.1016/s0031-9384(97)00539-8

[54] Wood LJ, Nail LM, Perrin NA, Elsea CR, Fischer A, Druker BJ. The cancer chemotherapy drug etoposide (VP-16) induces proinflammatory cytokine production and sickness behavior-like symptoms in a mouse model of cancer chemotherapy-related symptoms. Biol Res Nurs. 2006;8: 157–169. 10.1177/1099800406290932 17003255

[55] Carmichael MD, Davis JM, Murphy EA, Brown AS, Carson JA, Mayer EP, et al Role of brain IL-1beta on fatigue after exercise-induced muscle damage. Am J Physiol Regul Integr Comp Physiol. 2006;291: R1344–1348. 10.1152/ajpregu.00141.2006 16778069

[56] Chou TC, Lee CE, Lu J, Elmquist JK, Hara J, Willie JT, et al Orexin (hypocretin) neurons contain dynorphin. J Neurosci Off J Soc Neurosci. 2001;21: RC168. 10.1523/JNEUROSCI.21-19-j0003.2001 PMC6762880 11567079

[57] Schöne C, Burdakov D. Glutamate and GABA as rapid effectors of hypothalamic “peptidergic” neurons. Front Behav Neurosci. 2012;6: 81 10.3389/fnbeh.2012.00081 23189047 PMC3505835

[58] Wang XS, Woodruff JF. Cancer-related and treatment-related fatigue. Gynecol Oncol. 2014; 10.1016/j.ygyno.2014.10.013 PMC4355326 25458588

[59] Trotti LM, Bliwise DL. Treatment of the sleep disorders associated with Parkinson’s disease. Neurother J Am Soc Exp Neurother. 2014;11: 68–77. 10.1007/s13311-013-0236-z PMC3899483 24272458

[60] Sheng P, Hou L, Wang X, Wang X, Huang C, Yu M, et al Efficacy of modafinil on fatigue and excessive daytime sleepiness associated with neurological disorders: a systematic review and meta-analysis. PloS One. 2013;8: e81802 10.1371/journal.pone.0081802 24312590 PMC3849275

[61] Jean-Pierre P, Morrow GR, Roscoe JA, Heckler C, Mohile S, Janelsins M, et al A phase 3 randomized, placebo-controlled, double-blind, clinical trial of the effect of modafinil on cancer-related fatigue among 631 patients receiving chemotherapy. Cancer. 2010;116: 3513–3520. 10.1002/cncr.25083 20564068 PMC2941794

[62] Mendonça DA, Menezes K, Jog MS. Methylphenidate improves fatigue scores in Parkinson disease: a randomized controlled trial. Mov Disord Off J Mov Disord Soc. 2007;22: 2070–2076. 10.1002/mds.21656 17674415

[63] Minton O, Richardson A, Sharpe M, Hotopf M, Stone PC. Psychostimulants for the management of cancer-related fatigue: a systematic review and meta-analysis. J Pain Symptom Manage. 2011;41: 761–767. 10.1016/j.jpainsymman.2010.06.020 21251796

[64] Kerr CW, Drake J, Milch RA, Brazeau DA, Skretny JA, Brazeau GA, et al Effects of methylphenidate on fatigue and depression: a randomized, double-blind, placebo-controlled trial. J Pain Symptom Manage. 2012;43: 68–77. 10.1016/j.jpainsymman.2011.03.026 22208450

[65] Volkow ND, Wang G-J, Fowler JS, Logan J, Gerasimov M, Maynard L, et al Therapeutic Doses of Oral Methylphenidate Significantly Increase Extracellular Dopamine in the Human Brain. J Neurosci. 2001;21: RC121–RC121. 11160455 10.1523/JNEUROSCI.21-02-j0001.2001 PMC6763805

[66] Prommer E. Methylphenidate: established and expanding roles in symptom management. Am J Hosp Palliat Care. 2012;29: 483–490. 10.1177/1049909111427029 22144657

[67] Felger JC, Miller AH. Cytokine effects on the basal ganglia and dopamine function: the subcortical source of inflammatory malaise. Front Neuroendocrinol. 2012;33: 315–327. 10.1016/j.yfrne.2012.09.003 23000204 PMC3484236

[68] Capuron L, Pagnoni G, Drake DF, Woolwine BJ, Spivey JR, Crowe RJ, et al Dopaminergic mechanisms of reduced basal ganglia responses to hedonic reward during interferon alfa administration. Arch Gen Psychiatry. 2012;69: 1044–1053. 10.1001/archgenpsychiatry.2011.2094 23026954 PMC3640298

[69] Felger JC, Li L, Marvar PJ, Woolwine BJ, Harrison DG, Raison CL, et al Tyrosine metabolism during interferon-alpha administration: association with fatigue and CSF dopamine concentrations. Brain Behav Immun. 2013;31: 153–160. 10.1016/j.bbi.2012.10.010 23072726 PMC3578984

[70] Worden LT, Shahriari M, Farrar AM, Sink KS, Hockemeyer J, Müller CE, et al The adenosine A2A antagonist MSX-3 reverses the effort-related effects of dopamine blockade: differential interaction with D1 and D2 family antagonists. Psychopharmacology (Berl). 2009;203: 489–499. 10.1007/s00213-008-1396-0 19048234 PMC2875246

[71] Nunes EJ, Randall PA, Estrada A, Epling B, Hart EE, Lee CA, et al Effort-related motivational effects of the pro-inflammatory cytokine interleukin 1-beta: studies with the concurrent fixed ratio 5/ chow feeding choice task. Psychopharmacology (Berl). 2014;231: 727–736. 10.1007/s00213-013-3285-4 24136220 PMC4468782

[72] Schwartz N, Temkin P, Jurado S, Lim BK, Heifets BD, Polepalli JS, et al Chronic pain. Decreased motivation during chronic pain requires long-term depression in the nucleus accumbens. Science. 2014;345: 535–542. 10.1126/science.1253994 25082697 PMC4219555

[73] Gaykema RPA, Park SM, McKibbin CR, Goehler LE. Lipopolysaccharide suppresses activation of the tuberomammillary histaminergic system concomitant with behavior: a novel target of immune-sensory pathways. Neuroscience. 2008;152: 273–287. 10.1016/j.neuroscience.2007.10.042 18082968 PMC2562932

[74] Katafuchi T, Kondo T, Take S, Yoshimura M. Brain cytokines and the 5-HT system during poly I:C-induced fatigue. Ann N Y Acad Sci. 2006;1088: 230–237. 10.1196/annals.1366.020 17192569

[75] Eldadah BA. Fatigue and fatigability in older adults. PM R. 2010;2: 406–413. 10.1016/j.pmrj.2010.03.022 20656622

[76] Norden DM, Godbout JP. Review: microglia of the aged brain: primed to be activated and resistant to regulation. Neuropathol Appl Neurobiol. 2013;39: 19–34. 10.1111/j.1365-2990.2012.01306.x 23039106 PMC3553257

[77] Sorge RE, Mapplebeck JCS, Rosen S, Beggs S, Taves S, Alexander JK, et al Different immune cells mediate mechanical pain hypersensitivity in male and female mice. Nat Neurosci. 2015;18: 1081–1083. 10.1038/nn.4053 26120961 PMC4772157

[78] Bower JE, Ganz PA, Irwin MR, Arevalo JMG, Cole SW. Fatigue and gene expression in human leukocytes: increased NF-κB and decreased glucocorticoid signaling in breast cancer survivors with persistent fatigue. Brain Behav Immun. 2011;25: 147–150. 10.1016/j.bbi.2010.09.010 20854893 PMC3603145

[79] Lattie EG, Antoni MH, Fletcher MA, Penedo F, Czaja S, Lopez C, et al Stress management skills, neuroimmune processes and fatigue levels in persons with chronic fatigue syndrome. Brain Behav Immun. 2012;26: 849–858. 10.1016/j.bbi.2012.02.008 22417946 PMC3572196

[80] Clevenger L, Schrepf A, Christensen D, DeGeest K, Bender D, Ahmed A, et al Sleep disturbance, cytokines, and fatigue in women with ovarian cancer. Brain Behav Immun. 2012;26: 1037–1044. 10.1016/j.bbi.2012.04.003 22543257 PMC3434312

[81] Wang XS, Shi Q, Williams LA, Cleeland CS, Mobley GM, Reuben JM, et al Serum interleukin-6 predicts the development of multiple symptoms at nadir of allogeneic hematopoietic stem cell transplantation. Cancer. 2008;113: 2102–2109. 10.1002/cncr.23820 18792065 PMC2633777

[82] Schrepf A, Clevenger L, Christensen D, DeGeest K, Bender D, Ahmed A, et al Cortisol and inflammatory processes in ovarian cancer patients following primary treatment: relationships with depression, fatigue, and disability. Brain Behav Immun. 2013;30 Suppl: S126–134. 10.1016/j.bbi.2012.07.022 22884960 PMC3697797

